# Linkage mapping combined with GWAS revealed the genetic structural relationship and candidate genes of maize flowering time-related traits

**DOI:** 10.1186/s12870-022-03711-9

**Published:** 2022-07-08

**Authors:** Jian Shi, Yunhe Wang, Chuanhong Wang, Lei Wang, Wei Zeng, Guomin Han, Chunhong Qiu, Tengyue Wang, Zhen Tao, Kaiji Wang, Shijie Huang, Shuaishuai Yu, Wanyi Wang, Hongyi Chen, Chen Chen, Chen He, Hui Wang, Peiling Zhu, Yuanyuan Hu, Xin Zhang, Chuanxiao Xie, Xiaoduo Lu, Peijin Li

**Affiliations:** 1 The National Engineering Laboratory of Crop Resistance Breeding, School of Life Sciences, Anhui Agricultural University, Hefei, 230036 China; 2 School of Agronomy, Anhui Agricultural University, Hefei, 230036 China; 3 Institute of Crop Sciences, Chinese Academy of Agricultural Sciences, National Key Facility for Crop Gene Resources and Genetic Improvement, Beijing, 100081 China

**Keywords:** Maize, Flowering time, Quantitative trait locus, Genome-wide association study, Candidate gene

## Abstract

**Background:**

Flowering time is an important agronomic trait of crops and significantly affects plant adaptation and seed production. Flowering time varies greatly among maize (*Zea mays*) inbred lines, but the genetic basis of this variation is not well understood. Here, we report the comprehensive genetic architecture of six flowering time-related traits using a recombinant inbred line (RIL) population obtained from a cross between two maize genotypes, B73 and Abe2, and combined with genome-wide association studies to identify candidate genes that affect flowering time.

**Results:**

Our results indicate that these six traits showed extensive phenotypic variation and high heritability in the RIL population. The flowering time of this RIL population showed little correlation with the leaf number under different environmental conditions. A genetic linkage map was constructed by 10,114 polymorphic markers covering the whole maize genome, which was applied to QTL mapping for these traits, and identified a total of 82 QTLs that contain 13 flowering genes. Furthermore, a combined genome-wide association study and linkage mapping analysis revealed 17 new candidate genes associated with flowering time.

**Conclusions:**

In the present study, by using genetic mapping and GWAS approaches with the RIL population, we revealed a list of genomic regions and candidate genes that were significantly associated with flowering time. This work provides an important resource for the breeding of flowering time traits in maize.

**Supplementary Information:**

The online version contains supplementary material available at 10.1186/s12870-022-03711-9.

## Background

The floral transition is an important developmental trait for plant adaptation and reproduction and has been considered a critical selection criterion in crop breeding [1]. Over the past few decades, the major components and genetic pathways controlling maize flowering time have been preliminarily determined [2], and some important flowering time genes have been screened [3–5], but there are some challenges to study the flowering time of maize [6], and new regulatory sites still need to be uncovered.

Through comparisons with the flowering time homologous genes in model organisms such as *Arabidopsis thaliana* and rice (*Oryza sativa*), a series of factors controlling the flowering time of maize have been reported [7–10]. For instance, *Zea mays CENTRORADIALIS 8* (*ZCN8*), a homolog of *FLOWERING LOCUS T* (*FT*) in *Arabidopsis thaliana*, plays an important role in regulating maize flowering time, and the stepwise *cis*-regulatory variations within the gene promoter are essential for maize adaptation to local environment [11, 12]. The expression of *INDETERMINATE 1* (*ID1*) in immature leaves promotes the transition of maize flowering through the autonomous flowering pathway [13]. *DELAYED FLOWERING 1* (*DLF1*), a downstream component of the *ID1* signaling pathway, mediates the floregen signal transmission from the leaves to the shoot apex, which ensures the flowering transition of maize [7].

The flowering time variation in maize is accompanied by a change in leaf number, in addition to the silking and anthesis stages [6]. Compared with *Arabidopsis thaliana*, which display a consistent relationship between the number of leaves and the trait of days to flowering [14], the leaf number of maize has a more complex genetic context. Successive leaves are usually positioned at an angle of 180° to each other [15]. The leaves are initiated from the shoot apical meristem (SAM) in repeated phytomer units until the tassel terminates. The number of leaves or phytomers required for the vegetative growth stage is determined largely by the leaf initiation rate and the period of SAM reprogramming [6]. Analyses of maize mutants with differing leaf numbers have identified several genes that affect the leaf initiation rate or SAM reprogramming [16, 17]; for example, downregulated expression of *ZmMADS1,* an ortholog of *SUPPRESSOR OF CONSTANS* (*SOC1*) in *Arabidopsis*, results in delayed flowering and increased leaf numbers in maize [16]. Additionally, *UNBRANCHED 2* (*UB2*) and *UB3* encode SBP transcription factors that affect the inflorescence structure and the initiation rate of lateral leaf primordia, which in turn leads to an increase in leaf numbers [17].

The flowering time of maize exhibits tremendous natural diversity [18]. To identify the genetic factors that control the variation in maize flowering time, several populations have been constructed for the extensive mapping of quantitative trait loci (QTLs) [19–22]. A combination of bi-parental association populations and high-throughput sequencing technology was used to reveal that the flowering time of maize is controlled by complex genetic structures, for which numerous small-effect QTLs have been mapped [18, 23]. Although a few flowering time genes such as *Vegetative to generative transition 1* (*Vgt1*) and *ZmCCT* have been discovered, the resolution of the QTL analysis is often decreased by recombination events that occur in the mapping populations [24–26]. To alleviate this problem, genome-wide association studies (GWAS) based on genetic linkage disequilibrium (LD) were performed, which are advantageous for the detection of complex genetic variations; however, genotyping errors and population structure result in a large proportion of false positive or false negative results, interfering with the reliability of the mapping [27]. The robustness of linkage mapping and the high resolution of association mapping were therefore combined to enhance the power of the analysis. Using this joint approach, many candidate genes within the flowering time QTL intervals have been identified in maize [28–30].

Previous studies mainly focused on the genetic mapping of flowering time or leaf number in maize, and few studies have analyzed the relationship between flowering time and leaf number traits. In this study, we performed genetic structure analysis of flowering time and leaf number traits in 261 recombinant inbred lines (RILs) obtained from the cross between B73 and Abe2. We analyzed six flowering time-related traits: days to heading (DTH), days to silking (DTS), days to anthesis (DTA), number of leaves above the primary ear (LA), number of leaves below the primary ear (LB), and total leaf number (TLN). With these data, QTL analysis of maize flowering time was performed using a genetic map constructed by 10,114 polymorphic markers. Then we combined GWAS analysis of 244 different maize inbred lines with linkage mapping to further reveal candidate genes controlling flowering time in maize.

## Results

### Phenotypic variation and heritability of flowering time-related traits

The RIL population of B73 × Abe2 (designated BA), comprising 261 lines, was grown in Hainan (longitude 108.9°E, latitude 18.6°N) and Hefei (longitude 117.2°E, latitude 31.8°N), China, to assay flowering time-related traits under two distinct growth environments. The mean, range, standard deviation, broad-sense heritability values, and ANOVA results are presented in Table 1. Compared with B73, the Abe2 line flowered earlier. The flowering time difference between these two parental lines was larger in Hainan (8.50, 6.93, and 5.44 days for DTH, DTS, and DTA, respectively) than in Hefei (0.67, 1.29, and 1.33 days for DTH, DTS, and DTA) (Table 1, Fig. S1), suggesting that flowering time was influenced by the growth conditions in the two field trials. Both in Hainan and Hefei, the LA value was greater in B73 than in Abe2, in contrast to the trend observed for LB.

**Table 1:** Descriptive statistics, variance components, and broad-sense heritability of flowering time-related traits in the BA RIL population

Traits	Environment	B73 Mean ± SD	Abe2 Mean ± SD	Mean ± SD	Range	*G*	G*E	*H*^*2*^
DTH	Hainan	61.82 ± 2.86	53.32 ± 1.67	57.03 ± 3.01	48.13 — 66.38	60.74**		62.75%
	Hefei	69.17 ± 1.33	68.50 ± 2.17	66.10 ± 4.39	56.00 — 78.00	89.26**		60.82%
	Combined	64.41 ± 4.33	56.96 ± 6.85	61.56 ± 5.89	48.13 — 78.00	115.05**	33.77**	76.20%
DTS	Hainan	66.43 ± 1.90	59.50 ± 1.15	62.46 ± 3.09	54.25 — 70.75	64.63**		61.85%
	Hefei	73.00 ± 2.28	71.71 ± 1.70	71.72 ± 4.54	61.25 — 86.00	93.73**		59.25%
	Combined	69.46 ± 3.95	62.92 ± 5.74	67.08 ± 6.04	54.25 — 86.00	109.76**	47.98**	68.03%
DTA	Hainan	64.33 ± 1.80	58.89 ± 2.00	60.97 ± 2.78	53.83 — 69.75	51.43**		55.83%
	Hefei	71.33 ± 1.86	70.00 ± 0.76	70.14 ± 4.40	59.14 — 83.50	84.97**		58.08%
	Combined	67.13 ± 3.96	62.19 ± 5.44	65.54 ± 5.88	53.83 — 83.50	96.2**	42.17**	67.78%
LA	Hainan	5.67 ± 0.52	4.50 ± 0.52	5.00 ± 0.60	3.13 — 6.86	2.68**		50.22%
	Hefei	5.67 ± 0.62	4.50 ± 0.63	4.66 ± 0.63	3.00 — 7.13	2.01**		43.96%
	Combined	5.67 ± 0.58	4.50 ± 0.57	4.83 ± 0.64	3.00 — 7.13	3.37**	0.76**	79.94%
LB	Hainan	6.33 ± 0.82	6.94 ± 0.77	7.35 ± 0.87	4.75 — 10.00	5.31**		55.98%
	Hefei	5.67 ± 0.82	7.56 ± 0.81	6.56 ± 0.93	4.00 — 9.00	3.93**		49.73%
	Combined	5.86 ± 0.85	7.25 ± 0.84	6.96 ± 0.98	4.00 — 10.00	6.45**	2.28**	71.97%
TLN	Hainan	12.00 ± 1.10	11.50 ± 0.97	12.36 ± 1.13	8.25 — 15.38	8.96**		57.69%
	Hefei	11.33 ± 1.11	12.06 ± 1.06	11.19 ± 1.24	5.50 — 14.63	6.83**		53.34%
	Combined	11.52 ± 1.12	11.78 ± 1.04	11.78 ± 1.32	5.50 — 15.38	11.01**	3.55**	73.98%

*G* genotype, G*E genotype × environment interaction, *H*^*2*^ broad-sense heritability, DTH days to heading, DTS days to silking, DTA days to anthesis, LA number of leaves above the primary ear, LB number of leaves below the primary ear, TLN total leaf number

^**^Significance at *P* < 0.01

The RIL population exhibited a wide phenotypic variation in DTH, DTS, and DTA, as well as LA, LB, and TLN. Significant effects of genotype and environment × genotype (*G* × *E*) were detected for all flowering traits in the population (Table 1). Broad-sense heritability was used to estimate the heritability of flowering time and leaf number in the two different growth environments. Combined with the overall heritability of two different environmental effects, the broad-sense heritability of DTH, DTS and DTA were 76.20%, 68.03% and 67.78%, respectively, and the broad-sense heritability of LA, LB and TLN reached 79.94%, 71.97% and 73.98% (Table 1). These results suggest that the phenotypic variations of flowering time-related traits are mainly controlled by genetic factors, and the flowering time data are suitable for further QTL mapping.

### Correlation analysis of maize flowering time-related traits

**Fig. 1:**
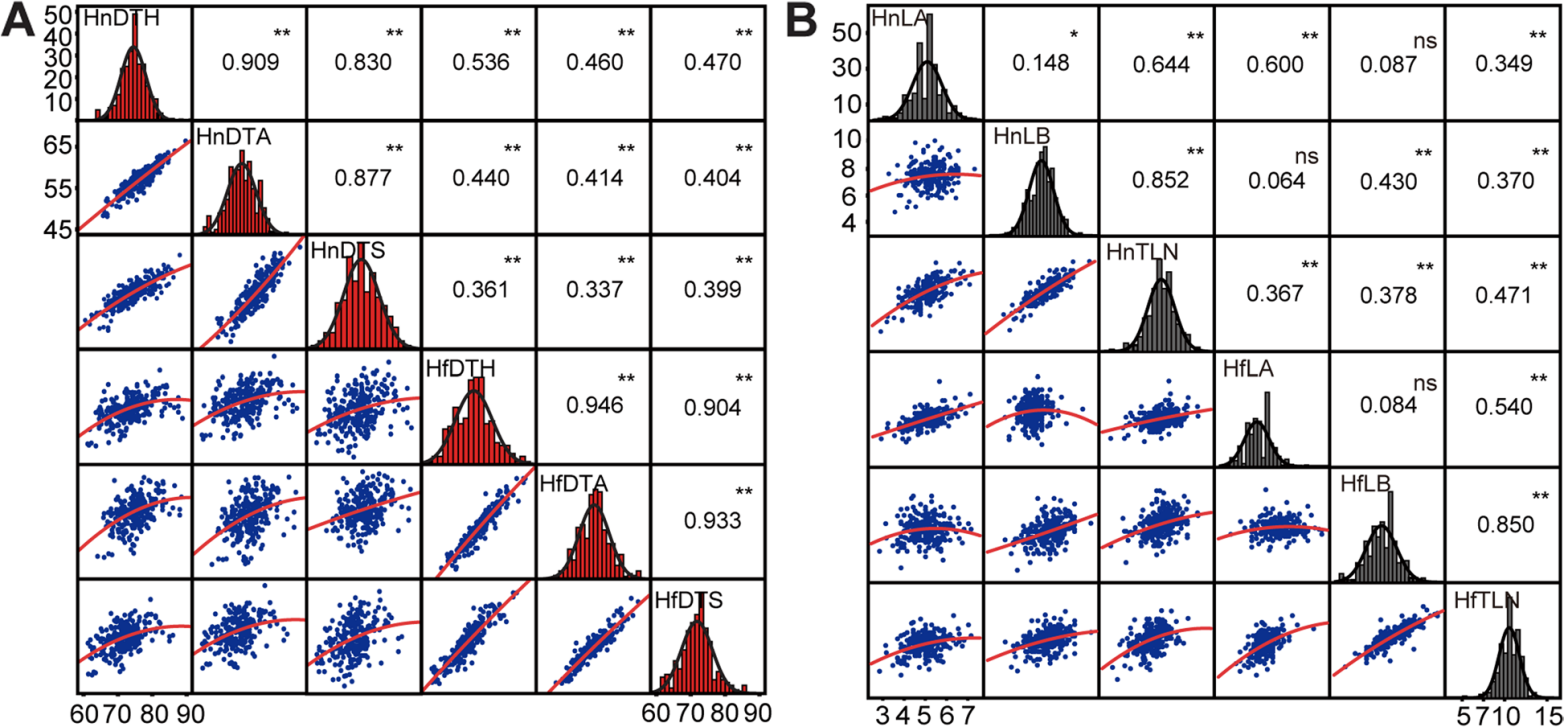
Correlation analysis of the flowering time-related traits in the BA population under different growth conditions. **A** Correlation analysis of flowering time traits. **B** Correlation analysis of leaf number traits. The normal distribution maps on the diagonals show the phenotypic distribution of each trait, as indicated. The values above the diagonal line are the pairwise correlation coefficients between the paired traits. The plots below the diagonal line are scatter plots of the compared traits. Significance: *, *P* ≤ 0.05; **, *P* ≤ 0.01; ns, not significant. DTH, days to heading; DTS, days to silking; DTA, days to anthesis; LA, number of leaves above the primary ear; LB, number of leaves below the primary ear; TLN, total leaf number. Hn and Hf represent the field trials performed in Hainan and Hefei, respectively, which are two locations in China with distinct climates. The numbers around the figures represent the flowering time (**A**) and leaf number (**B**)

In the BA population, the six flowering time-related traits showed continuous approximately normal distributions (Fig. 1). The correlations between DTH, DTS, and DTA reached a significance level of *P* < 0.01, with the *r* values ranging from 0.830 to 0.946, all of which were strongly positive, whereas the correlation of these traits between the different environments were moderately positive, with the *r* values ranging from 0.337 to 0.536 (Fig. 1A). This suggests that the timing of silking and anthesis were quite coordinated in the flowering initiation period but were greatly influenced by the growing environment. For the number of leaves, the correlation between different traits was relatively more variable. LA and LB were not well correlated in either growth environment, with *r* values of 0.084 and 0.148, respectively (Fig. 1B). The correlation between LB and TLN was higher than that of LA with TLN. We also compared DTH, DTS, and DTA with the leaf number traits and found that the correlation between them was significant except for three pairs (HfLB and HnDTH, HfLB and HnDTS, HfTLN and HnDTS, where Hn and Hf represent Hainan and Hefei, respectively), with relatively low *r* values ranging from 0.11 to 0.36 (Fig. S2). This suggests that flowering time and leaf number traits are weakly correlated and may be under relatively independent genetic controls.

### Construction of the genetic map for QTL mapping

**Fig. 2:**
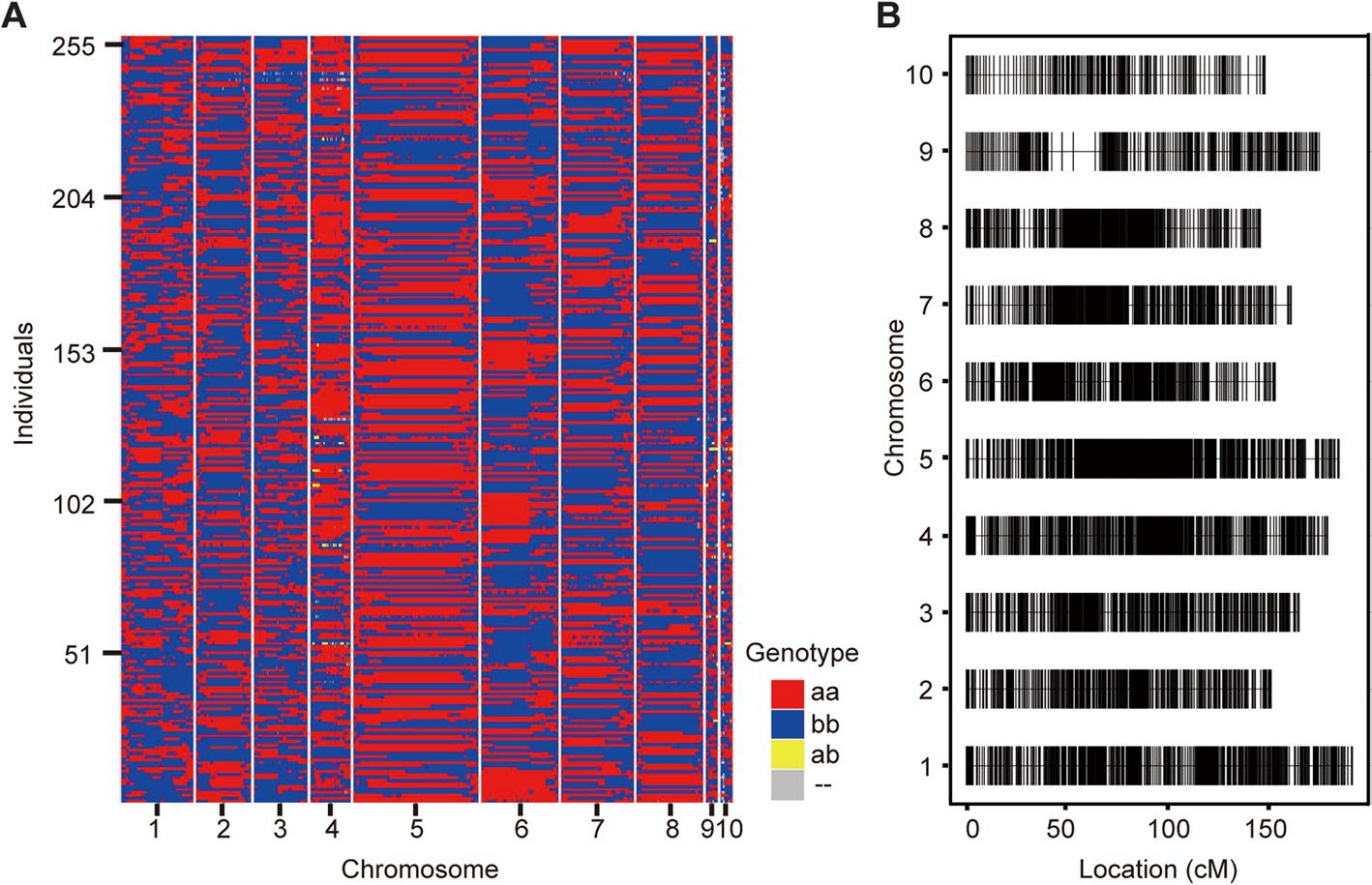
Genetic mapping of high-density markers and distribution of high-resolution genotyping markers in the BA population. **A** Recombinant bin map of the BA population. The horizontal axis represents the physical location of the single-nucleotide polymorphism (SNP) markers on each chromosome, and the vertical axis represents the 261 RILs. Red, Abe2 genotype (aa); blue, B73 genotype (bb); yellow, heterozygote (ab); gray, unknown genotype (–). **B** High-density genetic map of the BA population using SNP markers. The markers are represented by black bars. The horizontal axis represents genetic distance, and the vertical axis represents the 10 linkage groups

To map the QTLs for phenotypic variation, we constructed a genetic map with molecular markers. A total of 10,724 specific-locus amplified fragment (SLAF) sequencing markers were generated and assigned to 10 linkage groups (LGs). The modified logarithm of odds (MLOD) value was calculated using a pairwise tags exchange calculation, and the SLAF tags with MLOD values lower than 5 were removed. Finally, 10,114 markers were used for the genotype calling and were positioned on the map (Fig. 2A, B ; Table S1). The average integrity of all markers on the map reached 94.31%. This map covered a genetic distance of 1,657.6 cM, ranging from 146.06 to 191.76 cM for each linkage group, with an average marker interval of 0.16 cM (Table S1). LG 1 was the longest group with a coverage distance of 191.76 cM containing 1,206 loci, while LG 10 was the shortest group, spanning 148.32 cM and 218 loci. The map uniformity and the degree of linkage between markers were assessed as the percentage of ‘Gaps ≤ 5 cM’, which ranged from 98.74% to 100%. The largest gap of 10.9 cM was on LG 9 (Table S1).

To evaluate the quality of the genetic map, we performed a collinearity analysis by comparing the genetic position and physical position of each LG marker (Fig. S3). The results indicate that the genetic and genomic locations were collinear (Spearman *r* = 99.3–99.9% for each LG), which suggests that the accuracy of the calculated genetic recombination rate was high, and that the markers were suitable for subsequent QTL mapping.

### QTL mapping of flowering time-related traits

Table S2 summarizes the QTL mapping results and genetic structure of each trait under different growth conditions. The full list of QTLs for all traits is included in Table S3.

**Fig. 3:**
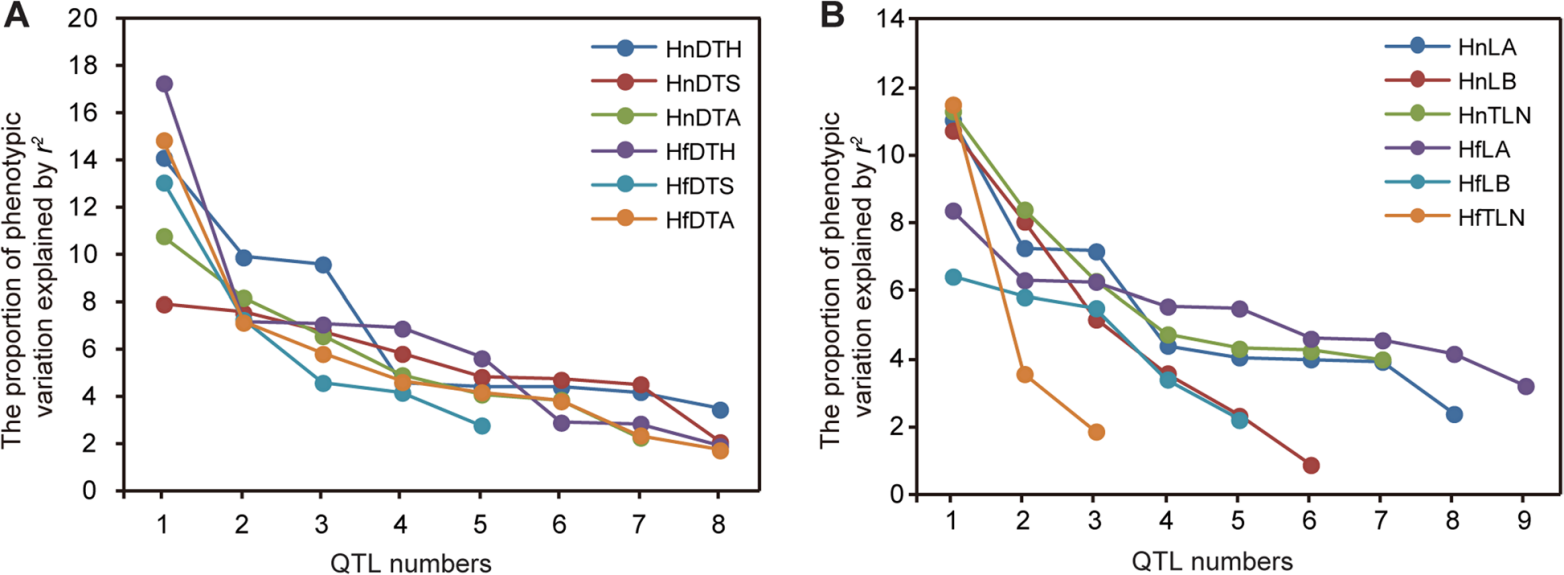
The distribution of phenotypic variation explained by each QTL. **A** Phenotypic variation distribution of flowering time traits in the BA population. **B** Phenotypic variation distribution of leaf number traits in the BA population. QTLs for each trait were ordered according to the proportion of phenotypic variation explained by *r*^*2*^. DTH, days to heading; DTS, days to silking; DTA, days to anthesis; LA, number of leaves above the primary ear; LB, number of leaves below the primary ear; TLN, total leaf number. Hn and Hf represent Hainan and Hefei, respectively, which are two locations in China with distinct climates

For DTH, eight QTLs were detected in Hainan and Hefei environments (Fig. 3A, Table S2). The phenotypic variation explained by each QTL ranged from 1.92% (*qHfDTH7-1*) to 17.28% (*qHfDTH9-1*) (Table S2). A major-effect QTL that explained more than 10% phenotypic variation, *qHnDTH9-1* (LOD = 7.15) and *qHfDTH9-1* (LOD = 11.53) in the population, was located on chromosome 9. The phenotypic variation explained by small-effect QTLs ranged from 1.92% to 9.91% and was evenly distributed (Fig. 3A, Table S2), indicating that the DTH trait was jointly controlled by the major- and small-effect QTLs.

A total of 13 QTLs were identified for DTS (Fig. 3A, Table S2), and the phenotypic variation in DTS explained by each QTL ranged from 2.09% (*qHnDTS3-1*) to 13.08% (*qHfDTS9-1*) (Table S2). The major-effect DTS QTL (*qHfDTS9-1*) only appeared in Hefei, with a LOD value of 8.68, but not in Hainan, suggesting that the effect of *qHfDTS9-1* on the silking stage in the BA population was greatly influenced by the environment.

For DTA, a total of 15 QTLs were mapped (Fig. 3A, Table S2). Each of these QTLs explained 2.28% (*qHnDTA1-1*) to 14.87% (*qHfDTA9-1*) of the total phenotypic variation (Table S2). A major-effect QTL was identified in each of the two growth conditions: *qHnDTA5-1* (LOD = 6.79) on chromosome 5 and *qHfDTA9-1* (LOD = 9.98) on chromosome 9.

The leaf number traits of the BA population were also subjected to the QTL analysis. The phenotypic variation explained by each QTL ranged from 0.89% (*qHnLB1-1*) to 11.50% (*qHfTLN8-1*) (Fig. 3B, Table S2). Major-effect QTLs for LA and LB (*qHnLA4-1* (LOD = 5.94) and *qHnLB7-1* (LOD = 7.19), respectively) were detected only in the Hainan trials, while many small-effect QTLs were identified in Hefei. Two different major-effect QTLs, *qHnTLN7-1* (LOD = 7.01) and *qHfTLN8-1* (LOD = 8.02), were associated with TLN in the two environments. These results further elucidate the complexity of the genetic structure underlying leaf number traits, which are also influenced greatly by the growing conditions.

### Flowering time genes were significantly enriched in the QTLs

**Fig. 4:**
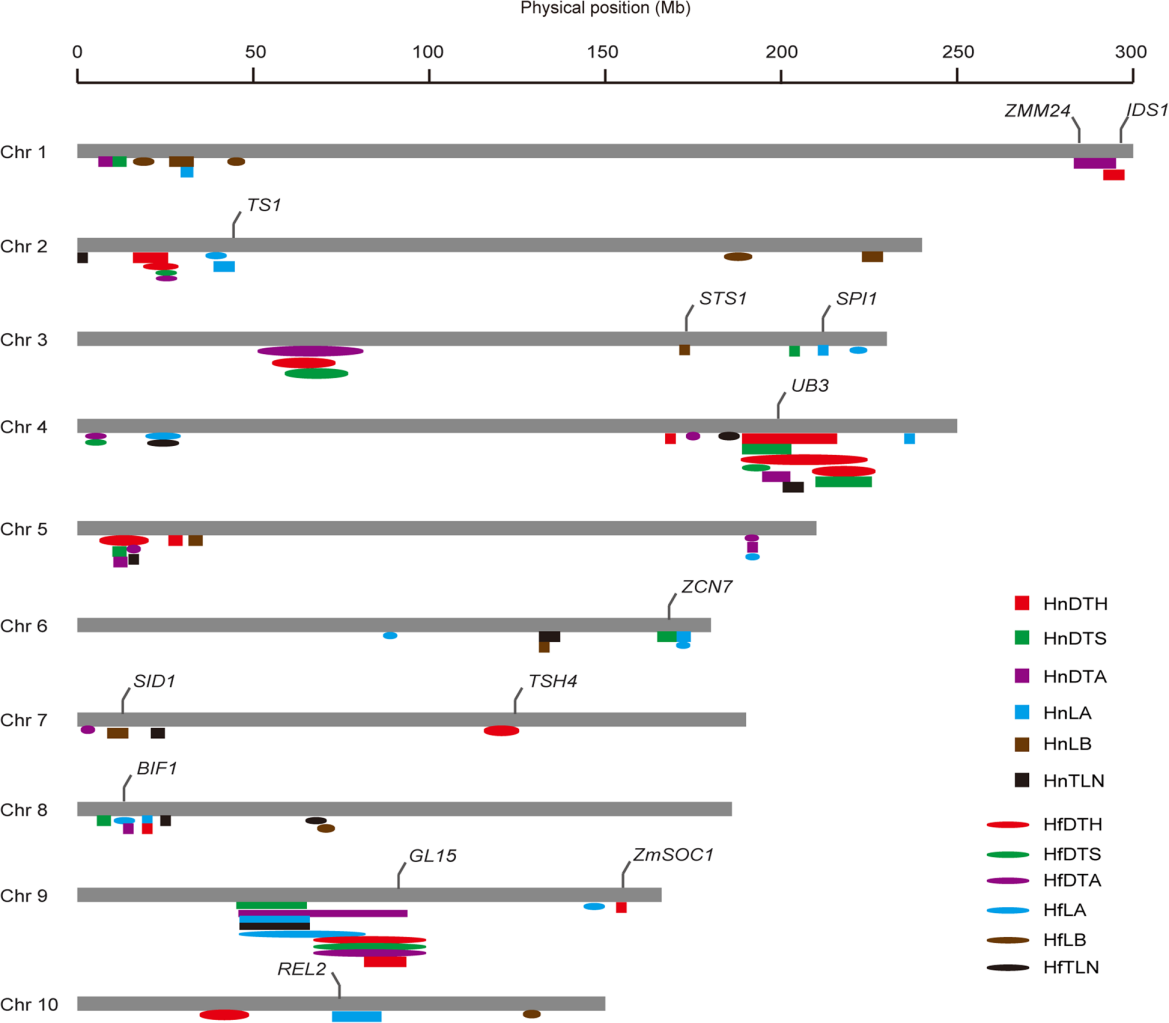
Genomic distribution of the QTLs for leaf number and flowering time traits The 10 chromosomes are shown as gray bars. Colored rectangles and ovals depict the 2-LOD support intervals for the QTLs identified in the BA population, as indicated. DTH, days to heading; DTS, days to silking; DTA, days to anthesis; LA, number of leaves above the primary ear; LB, number of leaves below the primary ear; TLN, total leaf number. Hn and Hf represent Hainan and Hefei, respectively, which are two locations in China with distinct climates. Previously reported genes are indicated above the chromosomes.

In previous studies, some of the genes such as *dlf1* and *ZmCCT* that controlling maize flowering time have been cloned through mutant analyses or QTL mapping [7, 31]. The mutation of these genes usually leads to changes in the silking and anthesis stages; therefore, we determined whether these flowering time genes were co-located with the QTLs in the BA population. We compared the 2-LOD support interval for these QTLs with 13 known flowering genes (Fig. 4, Table S4). A cluster of eight QTLs was located on chromosome 4, at a region containing a known maize flowering gene, *UB3* [17]. Another cluster on chromosome 9 contained nine QTLs overlapping *GLOSSY 15* (*GL15*), which is involved in maize flowering time control pathways [32]. Among the 13 flowering time genes co-located with the flowering time-related QTLs, only two genes, *UB3* and *GL15*, were located in the support interval of multiple flowering time-related QTLs, which may indicate that they play an important role on the control of flowering time variation.

### Candidate genes revealed by the combination of QTL mapping and GWAS

**Fig. 5:**
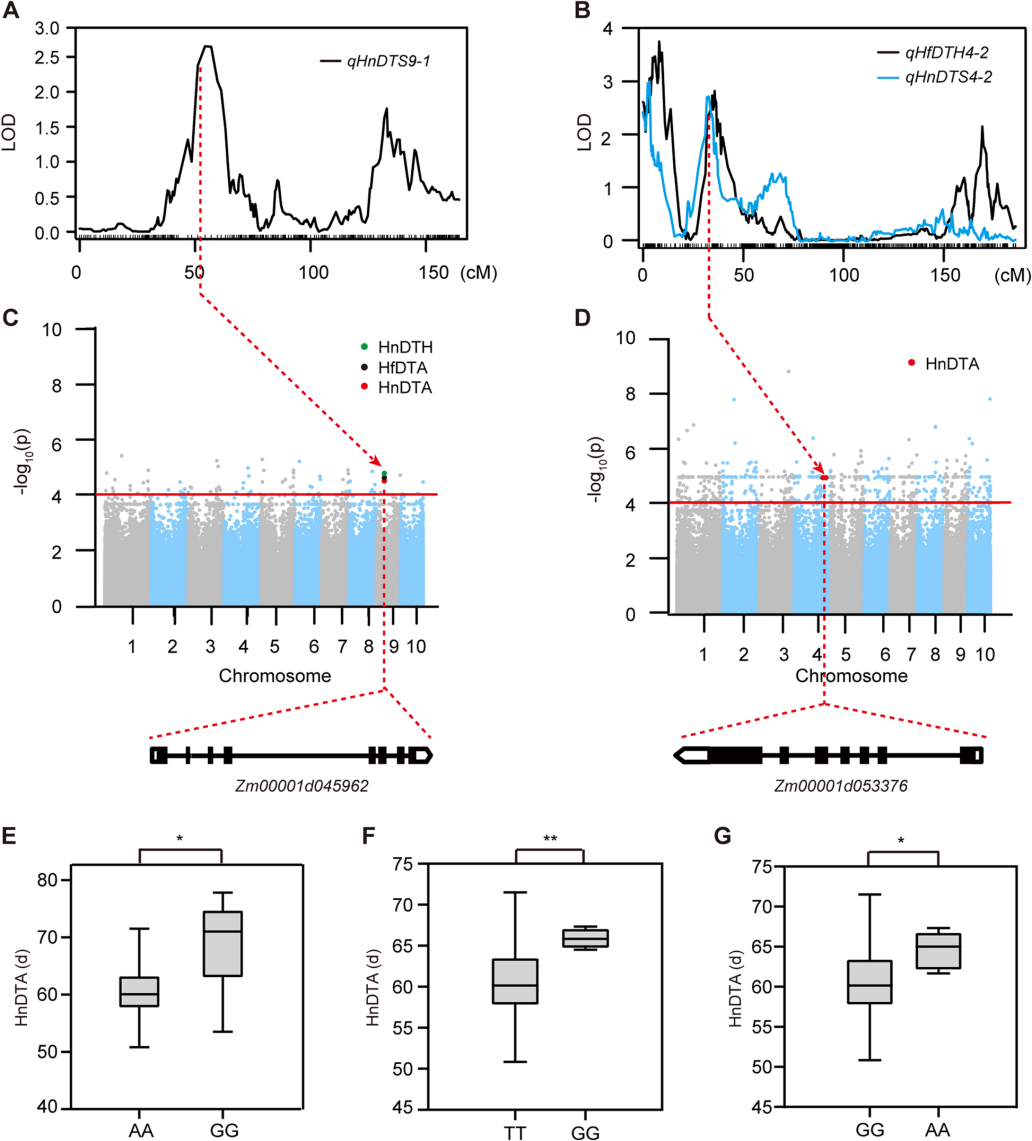
Combined QTL and GWAS analysis used to reveal the candidate genes. **A**–**B** QTLs for HnDTS on chromosome 9 (**A**), HfDTH and HnDTS on chromosome 4 (**B**). The red dashed line points to the candidate single-nucleotide polymorphism (SNP) location. **C**–**D** Manhattan plots of the GWAS analysis for HnDTH, HfDTA, HnDTA (**C**) and HnDTH (**D**) by mixed linear model (MLM). Horizontal dashed red line represents unified *P* value threshold. SNPs co-localized in the GWAS and QTL analyses are marked with larger dots. **E**–**G** Allelic effects of the significant SNPs on the *Zm00001d045962* (**E**) and *Zm00001d053376* (**F** and **G**) in HnDTA. DTA, days to anthesis. Significance: *, *P* ≤ 0.05; **, *P* ≤ 0.01

To narrow down the genomic region of the candidate genes, we performed GWAS analysis of flowering time traits using 244 maize inbred lines [33]. Combined with population structure and genetic relationship, SNP markers were identified by a mixed linear model (MLM), and 209 related SNPs (*P* < 1 × 10^–4^) were co-located with QTLs (Table S5). Among the 209 significantly associated SNPs, 47 and 162 were detected in Hefei and Hainan, respectively. Further analysis showed that 64 SNPs were located in at least 2 QTL intervals (Table S5). According to the position of SNP on chromosome, a total of 26 significant SNPs were found in 17 candidate gene intervals (Table 2). Gene ontology (GO) annotation was performed on 17 candidate genes, and most genes were found to be involved in growth, cell formation, and amino acid metabolism processes. One of the QTLs for DTS, *qHnDTS9-1* (Fig. 5A), contained the SNP chr9.S_ 48,271,409 (A/G,* p* = 2.31E − 05, 2.47E − 05 and 3.36E − 05; Fig. 5C), which was used to identify a candidate gene *Zm00001d045962*. The allelic effect of the SNP haplotype was investigated, and the average DTA associated with the G allele (68.5 d) was significantly different from that of the A allele (60.8 d) (Fig. 5E). *qHfDTH4-2* and *qHnDTS4-2* (Fig. 5B) contains the SNP chr4_224038847 (T/G, *p* = 1.07E − 05; Fig. 5D) and the SNP chr4_224038876 (G/A, *p* = 1.07E − 05; Fig. 5D), which was associated with the candidate gene *Zm00001d053376*, encoding an AAA-type ATPase family protein / ankyrin repeat family protein. The average HnDTA for the G alleles was 65.9 d, which was significantly higher than for the T alleles (60.7 d; Fig. 5F). Similarly, the average HnDTA of the G allele was 60.7 d, which was significantly lower than for the A alleles (64.6 d; Fig. 5G).

**Table 2:** Co-localization of candidate genes revealed using the combined GWAS and QTL analysis

Traits	QTL	SNP	Chr	Allele	*P* value	Gene	Annotation
HfDTA	qHfLB1-1	chr1.S_19944812	1	T/C	3.86E-05	Zm00001d028010	Conserved oligomeric Golgi complex subunit 8
HnDTA	qHnDTA1-1	chr1.S_12443338	1	A/C	4.38E-05	Zm00001d027752	SH2 domain protein B
HnDTA	qHnDTA1-1	chr1.S_12443395	1	C/T	5.17E-05	Zm00001d027752	SH2 domain protein B
HnDTA	qHnLB1-1	chr1.S_29513840	1	T/G	8.75E-05	Zm00001d028303	Branched-chain-amino-acid aminotransferase
HnDTA	qHnLB1-1	chr1.S_29517378	1	G/T	3.58E-05	Zm00001d028303	Branched-chain-amino-acid aminotransferase
HnDTA	qHnLB1-1	chr1.S_35770394	1	G/C	1.07E-05	Zm00001d028469	PGG domain-containing protein
HnDTA	qHnDTA1-2	chr1.S_283970301	1	C/T	3.92E-05	Zm00001d034317	Methyltransferase
HnDTA	qHnDTA1-2/qHnDTH1-1	chr1.S_286863933	1	G/A	2.39E-05	Zm00001d034406	25.3 kDa vesicle transport protein
HnDTA	qHnDTA1-2/qHnDTH1-1	chr1.S_286980178	1	T/C	1.07E-05	Zm00001d034409	Hydroxyproline-rich glycoprotein family protein
HnDTA	qHnDTA1-2/qHnDTH1-1	chr1.S_290115635	1	G/A	5.62E-05	Zm00001d034519	Putative acyl-activating enzyme 18 peroxisomal
HnDTH	qHnDTA1-2	chr1.S_283970301	1	C/T	3.15E-05	Zm00001d034317	Methyltransferase
HnDTH	qHnDTA1-2/qHnDTH1-1	chr1.S_286863933	1	G/A	1.29E-05	Zm00001d034406	25.3 kDa vesicle transport protein
HnDTH	qHnDTA1-2/qHnDTH1-1	chr1.S_286980295	1	G/A	6.52E-05	Zm00001d034409	Hydroxyproline-rich glycoprotein family protein
HnLA	qHnDTA1-2	chr1.S_283229666	1	A/G	1.37E-05	Zm00001d034276	Uncharacterized protein
HfDTH	qHnLA2-1	chr2.S_42789389	2	C/T	6.62E-05	Zm00001d003446	Gamma-glutamyltranspeptidase 1
HnDTA	qHnTLN2-1	chr2.S_1084283	2	A/G	8.87E-05	Zm00001d001798	Minichromosome instability12b
HnDTA	qHnTLN2-1	chr2.S_1084664	2	T/C	3.25E-06	Zm00001d001798	Minichromosome instability12b
HnLA	qHnLA2-1	chr2.S_44261048	2	G/A	8.97E-05	Zm00001d003499	E3 ubiquitin-protein ligase ATL6
HnDTA	qHfDTH4-2/qHnDTS4-2	chr4.S_224038847	4	T/G	1.07E-05	Zm00001d053376	AAA-type ATPase family protein / ankyrin repeat family protein
HnDTA	qHfDTH4-2/qHnDTS4-2	chr4.S_224038876	4	G/A	1.07E-05	Zm00001d053376	AAA-type ATPase family protein / ankyrin repeat family protein
HfDTA	qHfDTH5-1	chr5.S_18632762	5	C/G	3.53E-05	Zm00001d013745	Uncharacterized protein
HnLB	qHfDTH5-1	chr5.S_17862164	5	T/A	9.68E-05	Zm00001d000202	Uncharacterized protein
HnLB	qHnDTS6-1	chr6.S_163425227	6	G/C	2.65E-05	Zm00001d038954	2-oxoglutarate and Fe (II)-dependent oxygenase superfamily protein
HfDTA	qHnDTS9-1	chr9.S_48271409	9	A/G	2.47E-05	Zm00001d045962	Uncharacterized protein
HnDTA	qHnDTS9-1	chr9.S_48271409	9	A/G	2.31E-05	Zm00001d045962	Uncharacterized protein
HnDTH	qHnDTS9-1	chr9.S_48271409	9	A/G	3.36E-05	Zm00001d045962	Uncharacterized protein

## Discussion

Flowering time is an essential agronomic trait for maize, and revealing its underlying molecular basis will enhance its yield and application in crop breeding. In the BA population, flowering time traits showed extensive phenotypic variation with a normal distribution and were highly heritable across the different environments (Fig. 1). *G* × *E* interaction effect has important value for agricultural breeding, and it reflects trait variation that cannot be explained by individual *G* and *E* effects [34, 35]. In this study, the *G* × *E* interaction effect of flowering time-related traits was significant (Table 1), which would facilitate the selection of stable varieties for breeding in different growth environments.

DTH, DTS, and DTA were all strongly positively correlated under the different environments, suggesting the internal coordination of flowering time to ensure consistent growth and development in maize plants (Fig. 1A). The maize TLN can be partitioned into two components, LA and LB, which are important in the breeding of plant architecture. An optimum proportion of LA and LB could improve photosynthetic potential and thereby increase maize yields [36]. Our data showed that LA and LB share a very low correlation (Fig. 1B), consistent with observations in other maize populations [37]. This suggests relative genetic independence between LA and LB and a complex genetic basis of maize leaf development. We also analyzed the relationship between leaf number and flowering traits in the BA population, and the results showed that there was little correlation between them (Fig. S2). This result implies that leaf growth and inflorescence development are controlled by relatively independent genetic pathways, consistent with previous observations [37].

As the number of recombination events increases, the resolution of QTL mapping can be improved using high-density genetic markers [38]. In this study, we constructed a genetic map with 10,114 markers. And a total of 82 QTLs associated with flowering time-related traits were detected. With the exception of HnDTS, HfLA, and HfLB, one major-effect QTL and multiple small-effect QTLs were identified for all other flowering time-related traits (Table S2). Some of these QTLs show genetic overlap (Fig. 4); for example, a cluster containing eight QTLs was detected on chromosome 4, and the overlap interval included not only QTLs for DTH, DTS, and DTA, but also QTLs for leaf number traits. Another cluster mapped on chromosome 9 also contained overlapping QTLs for flowering time traits, which reflects that the overlapped QTLs of different traits may play an important role in controlling maize flowering time (Fig. 4). Moreover, this QTL overlapping of multiple flowering time-related traits suggested that these phenotypes could be controlled by the same gene, but we cannot rule out the possibility that the causative genes for different traits cluster together in one QTL region, which provides a useful basis for fine-mapping and gene isolation in the future.

The flowering time of maize is mainly determined by the transition of the SAM from the initiation of leaf primordia to floral primordia. Delayed floral transition mutants produce more leaves owing to the extended leaf initiation period, as observed for several previously cloned genes in maize [7, 13]. The transition from young leaves to mature leaves is regulated by the *APETALA2*-like gene *GL15* in maize [32]. Increasing the activity of *GL15* not only increases the number of leaves in the maize juvenile phase but also delays the initiation of reproductive development, indicating that *GL15* plays an important role in maintaining the juvenile phase [32]. Here we found that the QTL on chromosome 9 encompasses *GL15* and is associated with flowering time traits (Fig. 4). It may be possible that the causative genes for this QTL are *GL15*-linked variations.

The genes that influence leaf initiation rate and pattern do so by changing the spatial and temporal patterns. The number of leaves usually depends on their initiation rate. For example, *TERMINAL EAR 1* (*TE1*) plays an important role in the early stages of leaf development. Mutations in *TE1* resulted in higher leaf initiation frequency, shorter internodes, and ultimately more leaves [39]. The initiation rate of lateral primordia is jointly regulated by the functionally redundant SBP transcription factors UB2 and UB3, and the double mutant *ub2 ub3* exhibited increased leaf number but showed no difference in flowering time [17]. This inconsistency may reflect the genetic control of leaf number and flowering time, which are closely related but relatively independent from each other. This phenomenon was also shown in our study, where the correlation between leaf number and flowering time phenotypes was low (Fig. S2). The floral transition is induced by leaf-derived signals, which translocate through the phloem to the shoot apex to reprogram the SAM [6]. However, it is currently unclear how this signal is coordinated given that leaf initiation and flowering time are not well synchronized (Fig. S2). This need to be addressed in future research.

GWAS approaches have been extensively applied to reveal the genetic basis for phenotypic variation in maize [22, 40]. By utilizing the high resolution of GWAS and robustness of QTLs, candidate genes in QTL regions were screened and verified [30, 41]. In this study, we applied this GWAS–QTL approach and identified seventeen candidate genes associated with flowering time and leaf number phenotypes (Table 2). *Zm00001d028303* is predicted to encode a branched-chain-amino-acid aminotransferase and co-located on chromosome 1. *Zm00001d028303* is a homolog of the *BRANCHED-CHAIN AMINOTRANSFERASE 3* (*BCAT3*) gene in *Arabidopsis thaliana*. The triple mutants (*bcat3*/*bcat4*/*bcat6*) exhibited smaller plants and reduced leaves [42]. Here, we found that chr9.S_ 48,271,409 located in *qHnDTS9-1* in both environments, and the related candidate gene *Zm00001d045962* encodes an unknown protein. The *Arabidopsis* homologs *OTS1*/*OTS2* of *Zm00001d045962* are involved in mediating the reversible SUMOylation of phyB, which in turn regulates plant flowering time through the photoperiodic pathway [43]. Therefore, *qHnDTS9-1* may be an important QTL and play a key role in flowering time. Another candidate gene *Zm00001d053376* was found in *qHfDTH4-2* and *qHnDTS4-2*, co-localizing with the GWAS results of HnDTA. The *Arabidopsis* homologous gene *XB3 ORTHOLOG 1 IN ARABIDOPSIS THALIANA* (*XBAT31*) of *Zm00001d053376* interacts with *EARLY FLOWERING3* (*ELF3*). Overexpression of *XBAT31* accelerates the degradation of *ELF3*, promotes the growth of hypocotyl and leads to early flowering [44]. The candidate genes, identified in this study, have SNP markers closely associated with phenotypes. Superior alleles and fine traits can be integrated in these varieties through molecular marker-assisted selection, which provides extensive genetic resources for future maize breeding.

## Conclusions

In this study, a RIL population genetic map was generated to analyze the flowering time-related traits of maize, and a total of 82 QTLs were identified. Furthermore, the combination of linkage analysis and GWAS was used to analyze the genetic structure of six traits related to flowering time in maize under two different growth environments, and 17 candidate genes were screened out. This work provides a valuable resource and will aid the molecular marker-assisted breeding of maize.

## Materials and methods

### Plant materials, field trials, and phenotyping

Two maize accessions, B73 and Abe2, were selected to construct a RIL population (named the BA population) as described previously [45]. A total of 261 stable RILs were used in the current research. The individual plants of the BA population were grown in fields located in Hainan (Hn; longitude 108.9°E, latitude 18.6°N), China, in 2017, and in Hefei (Hf; longitude 117.2°E, latitude 31.8°N), China, in 2018. Two parental lines, B73 and Abe2, were randomly planted together with the RILs for phenotypic comparison.

All of the lines of BA RIL population were grown randomly in each field location [46]. For each RIL line, 13 individuals were planted in fields and subjected to normal agricultural maintenance. The distance between plants within each row was 30 cm, and the distance between rows was 60 cm. The DTH, DTS, and DTA for all the individual plants from each line were recorded and averaged for further analysis. The LA and LB values of each plant were calculated independently. The TLN was the sum of LA and LB.

The GWAS population of 244 maize inbred lines (provided by Professor Jianbing Yan, Huazhong Agricultural University, Wuhan, China) was planted in both Hefei and Hainan in 2016. The field trials were carried out as described for the BA population above. Study complied with local and national regulations for using plants.

### Statistical analyses

Frequency distribution, correlation, and variance analyses were performed using SPSS Statistics 16.0 software (IBM, Armonk, New York, USA). R version 3.1.1 (www.R-project.org) was used for the statistical analysis of all traits, and the following mixed linear model was used for the multi-position test analysis to obtain the best linear unbiased predictor:

$$Y_{ijk} \, = \,\mu \, + \,E_{i\,} + rep(E)_{ij} \, + G_{k} + E + G_{jk} + \varepsilon_{ijk}$$

where *μ* is the grand mean of flowering traits, E_*i*_ is the environmental effect of the *i*th environment, rep(E)_*ij*_ is the effect of the *j*th replication within the *i*th environment, G_*k*_ is the genetic effect of the *k*th line, E × G_*ik*_ is the interaction between environmental and genetic effects, and ε_*ijk*_ is the residual error containing all the above experimental factors. All terms except the mean were fitted as random effects. All variance components were used to calculate the broad-sense heritability using the methods reported previously, as described previously [47]:

$${H}^{2}=\frac{{\upsigma }_{G}^{2}}{ [{\upsigma }_{G}^{2}+({\upsigma }_{GE}^{2} /\mathrm{n}) + ({\upsigma }_{e}^{2} /nr)]}$$

where ${\upsigma }_{GE}^{2}$ is the *G* × *E* variance, ${\upsigma }_{G}^{2}$ is the genetic variance, ${\upsigma }_{e}^{2}$ is the residual error, and *n* and *r* are the number of environments and the number of replications, respectively.

### DNA extraction and high-throughput sequencing

Young and healthy maize leaves were collected from the two parental lines and 261 RILs at the seedling stage, frozen in liquid nitrogen, and stored at –80 °C. Total genomic DNA was extracted using the cetyltrimethylammonium ammonium bromide (CTAB) method [48]. All samples with a suitable concentration and quality were used for library construction.

The library was constructed using the SLAF sequencing method described previously [49]. Two restriction enzymes, *Hpy*166II and *Hae*III (New England Biolabs, Ipswich, Massachusetts, USA), were selected to digest the genomic DNA into 414- to 464-bp fragments. The ends of completely digested fragments were repaired into blunt-ended DNA and the 5' ends were phosphorylated. An adenine base was added to the 3' end of the fragment, after which the duplex index sequencing adapter was connected to the A-tailed fragment. The target fragment was amplified, purified, and sequenced on an Illumina HiSeq 2500 platform (Illumina Inc., San Diego, California, USA).

### Construction of genetic map

The sequencing reads were aligned to the v4 reference genome using BWA software [50], and the SLAF markers were identified and genotyped. To ensure the quality of the genetic map, the polymorphic SLAF tags were removed before the map was constructed. HighMap software [51] was used to analyze the linear arrangement of markers in the linkage group and to estimate the genetic distance between adjacent markers. First, the recombination rate and the modified logarithm of odds (MLOD) value between tags were calculated, and then tags with MLOD values less than 5 were filtered. The molecular markers were divided into different linkage groups according to the MLOD value, and each linkage group was considered as a chromosome. Chromosomes were used as units to construct the genetic map using the maximum likelihood method. Finally, a complete genetic map of 1,657.6 cM was constructed.

### QTL mapping

A composite interval mapping analysis was carried out using R/qtl software for QTL mapping [52]. The threshold of the QTL value was determined at a significance level of *P* < 0.05 after 1,000 permutations. To investigate the genetic overlap between each trait, the 2-LOD support intervals of QTL were compared; QTLs with overlapping support intervals were regarded as common QTLs for the compared traits.

### GWAS

The flowering time trait phenotypes of the 244 maize inbred lines were analyzed using a GWAS approaches [33]. All inbred lines have been genotyped by MaizeSNP50 BeadChip maize array (Illumina) [53] and 556,809 high-quality SNPs were obtained by RNA sequencing (minor allelic frequencies, MAF ≥ 0.05) [54]. GWAS analysis was performed on both environments using a mixed linear model (MLM) in tassel version 5.0, which considered both population structure (Q) and kinship matrix (K) [55]. A uniform Bonferroni correction threshold of α = 1 for the MLM was used as the critical value of significance. Because the Bonferroni correction (1/*n* = 1.8 × 10^−6^, *n* = total markers used) was too conservative (there were few SNPs significantly associated with the six traits), a less stringent threshold of 1 × 10^−4^ was taken as the final significance threshold [56–58]. To reveal the unique candidate genes behind the associated signals, a LD analysis was performed on important SNPs on the same chromosome, with the LD statistic *r*^2^ set to > 0.2. The physical location of the SNP was determined based on version 2 of the maize genome sequence (https://www.maizegdb.org).

### Annotation of candidate genes

To reveal the correlation signal between the GWAS and QTL analyses, only the SNPs in QTL intervals supported by a LOD greater than 2.0 were considered to be overlapping SNPs. As in previous studies [53], the genes within 50 kb upstream or downstream of the SNP were explored as candidates regulating flowering time.

## Supplementary Information

**Additional file 1: Fig. S1.** The phenotype of theB73 and Abe2 inbred lines at the flowering stage inHainan. Thered arrows point to the tassel and ear of abe2, respectively. Scale bar= 20 cm. **Fig. S2.** Correlation coefficients of floweringtime-related traits in different field trials. The heat map representsthe correlation strength (*r*) between the paired traits.Significance: *, *P* ≤ 0.05; **, *P* ≤ 0.01. DTH, days to heading; DTS,days to silking; DTA, days to anthesis; LA, number of leaves above the primaryear; LB, number of leaves below the primary ear; TLN, total leaf number. Hn andHf represent Hainan and Hefei, respectively, which are two locations in Chinawith distinct climates. **Fig. S3.** Correlation between the genetic and physicallocations of the polymorphic specific-locus amplified fragment markers.

**Additional file 2: Table S1.** Summary of thehigh-density genetic map. **Table S2.** Summary of QTLs identified forflowering time-related traits in the BA population. DTH, days to heading; DTS, days to silking; DTA,days to anthesis; LA, number of leaves above the primary ear; LB, number ofleaves below the primary ear; TLN, total leaf number. Hn and Hf representHainan and Hefei, respectively. **Table S3.** QTLs for the leaf number and flowering time traits identified in the BApopulation. **Table S4.** List of known flowering time-related genesdetected in the QTL analysis. * Thephysical positions of genes are defined according to the maize B73 referencegenome AGPv4. **Table S5.** Significant SNP loci obtained by co-localizationof QTL and GWAS for flowering time traits.

## Data Availability

The datasets necessary for supporting the results of this manuscript are included in this manuscript (and its additional files). The data set generated by sequencing the BA population in this study are available at National Center for Biotechnology Information (NCBI) (accession number: PRJNA824290). The SNP data set for GWAS is available from http://www.maizego.org/Resources.html.

## Abbreviations

RIL: Recombinant inbred line
QTLs: Quantitative trait loci
SAM: Shoot apical meristem
GWAS: Genome-wide association studies
LD: Linkage disequilibrium
SNP: Single-nucleotide polymorphism
MAF: Minor allele frequency (MAF)
DTH: Days to heading
DTS: Days to silking
DTA: Days to anthesis
LA: Number of leaves above the primary ear
LB: Number of leaves below the primary ear
TLN: Total leaf number
MLM: Mixed linear model
SLAF: Specific-locus amplified fragment
LGs: Linkage groups
MLOD: Modified logarithm of odds
