# Supplementary material for: Linkage mapping combined with GWAS revealed the genetic structural relationship and candidate genes of maize flowering time-related traits
Source: BMC Plant Biol. 2022 Jul 8;22:328. doi: 10.1186/s12870-022-03711-9 (PMC9264602; doi:10.1186/s12870-022-03711-9)
Supplement: Supplementary file 1 — Additional file 1: Fig. S1. The phenotype of theB73 and Abe2 inbred lines at the flowering stage inHainan. Thered arrows point to the tassel and ear of abe2, respectively. Scale bar= 20 cm. Fig. S2. Correlation coefficients of floweringtime-related traits in different field trials. The heat map representsthe correlation strength (r) between the paired traits.Significance: *, P ≤ 0.05; **, P ≤ 0.01. DTH, days to heading; DTS,days to silking; DTA, days to anthesis; LA, number of leaves above the primaryear; LB, number of leaves below the primary ear; TLN, total leaf number. Hn andHf represent Hainan and Hefei, respectively, which are two locations in Chinawith distinct climates. Fig. S3. Correlation between the genetic and physicallocations of the polymorphic specific-locus amplified fragment markers. [file 12870_2022_3711_MOESM1_ESM.docx]

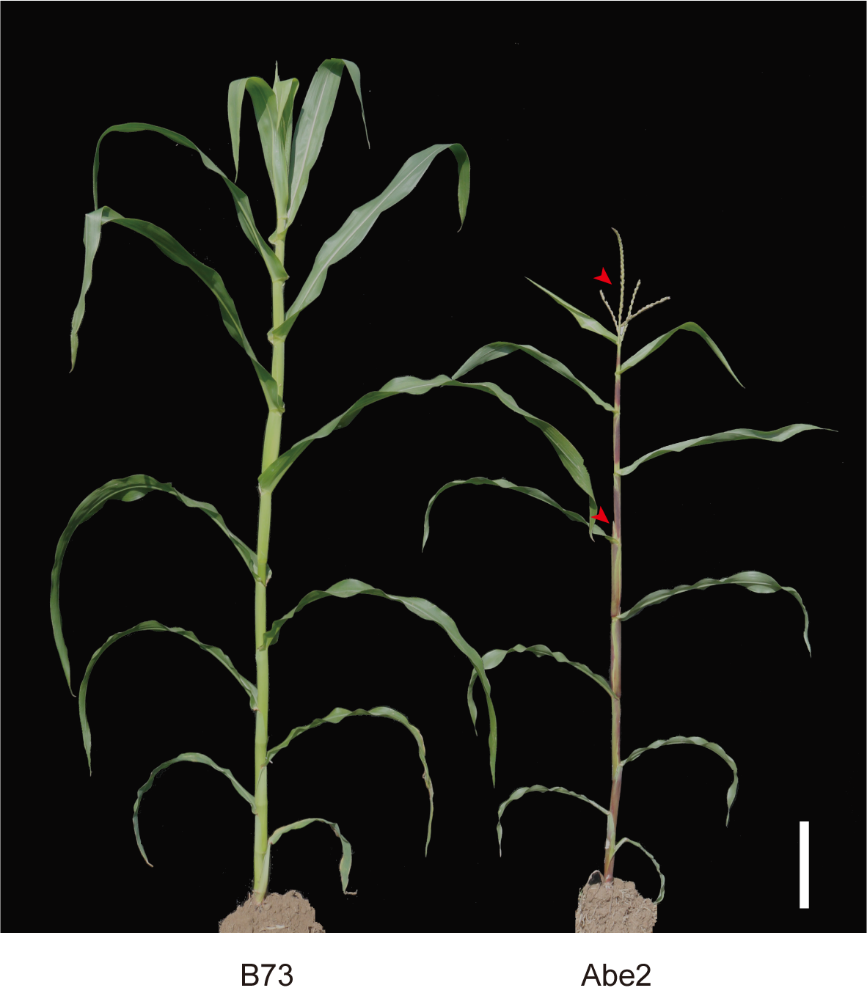


**Fig. S1 The phenotype of the B73 and Abe2 inbred lines at the flowering stage in Hainan.**

The red arrows point to the tassel and ear of abe2, respectively. Scale bar = 20 cm.


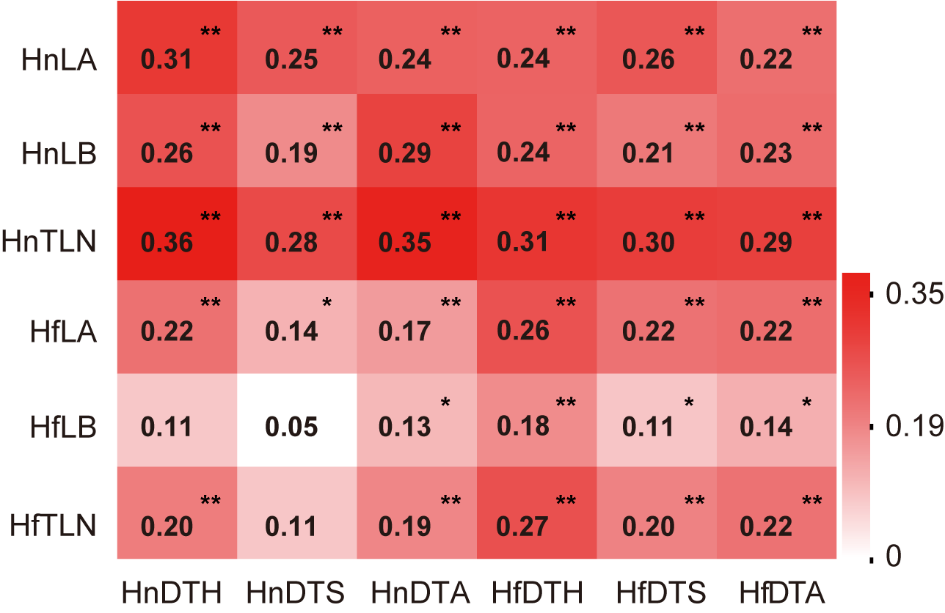


**Fig. S2 Correlation coefficients of flowering time-related traits in different field trials.**

The heat map represents the correlation strength (*r*) between the paired traits. Significance: *, *P* ≤ 0.05; **, *P* ≤ 0.01. DTH, days to heading; DTS, days to silking; DTA, days to anthesis; LA, number of leaves above the primary ear; LB, number of leaves below the primary ear; TLN, total leaf number. Hn and Hf represent Hainan and Hefei, respectively, which are two locations in China with distinct climates.


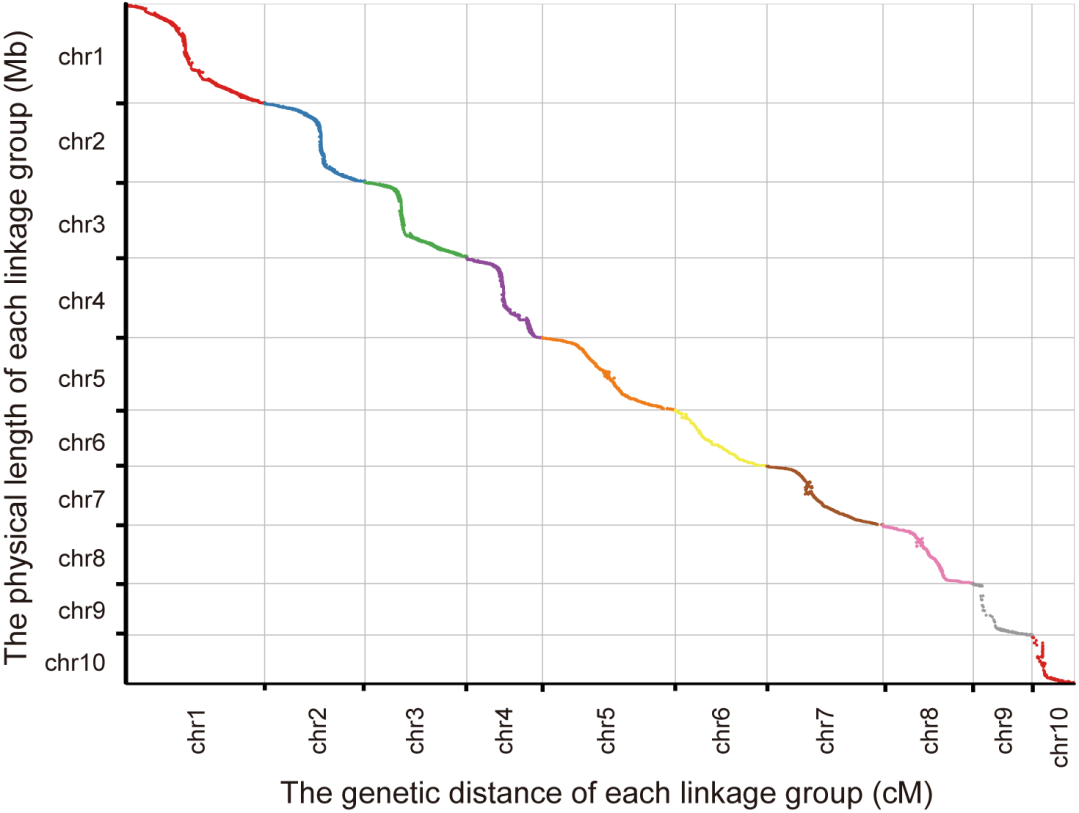


**Fig. S3 Correlation between the genetic and physical locations of the polymorphic specific-locus amplified fragment markers.**
